# An Investigation of Racing Performance and Whip Use by Jockeys in Thoroughbred Races

**DOI:** 10.1371/journal.pone.0015622

**Published:** 2011-01-27

**Authors:** David Evans, Paul McGreevy

**Affiliations:** Faculty of Veterinary Science, University of Sydney, Sydney, New South Wales, Australia; Indiana University, United States of America

## Abstract

Concerns have been expressed concerning animal-welfare issues associated with whip use during Thoroughbred races. However, there have been no studies of relationships between performance and use of whips in Thoroughbred racing. Our aim was to describe whip use and the horses' performance during races, and to investigate associations between whip use and racing performance. Under the Australian Racing Board (ARB) rules, only horses that are in contention can be whipped, so we expected that whippings would be associated with superior performance, and those superior performances would be explained by an effect of whipping on horse velocities in the final 400 m of the race. We were also interested to determine whether performance in the latter sections of a race was associated with performance in the earlier sections of a race. Measurements of whip strikes and sectional times during each of the final three 200 metre (m) sections of five races were analysed. Jockeys in more advanced placings at the final 400 and 200 m positions in the races whipped their horses more frequently. Horses, on average, achieved highest speeds in the 600 to 400 m section when there was no whip use, and the increased whip use was most frequent in the final two 200 m sections when horses were fatigued. This increased whip use was not associated with significant variation in velocity as a predictor of superior placing at the finish.

## Introduction

The tradition of whip use in racing is ancient and reflects a time when any intervention on animals, however painful, could be justified if humans benefited. Times have changed and concerns surrounding the justification for whipping horses are increasingly being vocalised by lay and industry commentators [1], [2]. The arguments against moderation in whip use are that whip use at the discretion of the jockey allows horses to give of their best.

Across the various sports and equestrian codes, there is a conspicuous absence of any real consensus on equine welfare indicators, even though almost all equestrian and racing governing bodies insist that horse welfare is of paramount importance. Various regulations govern the use of the whip in racing. For example, the British Horseracing Authority (BHA) and the Australian Racing Board (ARB) provide detailed instructions on the use of the whip in racing [3], [4]. In a bid to ensure that the whip is used for “safety, correction and encouragement only”, the ways in which the whip may be used and/or should not be used according to BHA and ARB rules are described. However, this is in the context of penalties for any jockey who fails to ride his horse out (i.e., ensures that the horse gave of its best) to the end of the race and/or approaching the end of the race (e.g., Rule137(b) in the ARB Rules of Racing [4]).

Padded whips are now required in both Australian and the UK and there are clear rules about how these can and cannot be used. However, for these devices to be effective they must activate mechanoreceptors in the horse's skin and, despite the padding, deformation of tissues remains a consequence. Breakable whips have been suggested as a useful means of placing an upper limit on this variable, but it seems obvious that if all horses were trained to gallop without whips, there would still be winners. It is important to note here that Norwegian racing authorities have taken a lead by banning the use of the whip as an accelerator in racing.

The role of learning theory in understanding a horse's responses to being whipped seems to be poorly appreciated by jockeys (and trainers) when horses are hit as they accelerate or when they cannot accelerate [5]. This equates to acceleration being punished and should result in it being inhibited. It has been argued that the only acceptable use of the whip would be for acceleration to be negatively reinforced by a mild persistent tapping action, where the frequency rather than the intensity of the tapping is increased, but ceases at the onset of the correct response [6].

The response of the horse to the whip cannot be assumed from the current literature, which is equivocal and circumstantial. However, a study of racing Quarter horses at the gallop showed that the use of a whip on the shoulder of the leading forelimb, in rhythm with the stride, reduced stride length and increased stride frequency without increasing speed [7].

There have been no peer-reviewed studies of relationships between performance and use of whips during Thoroughbred races. We do not know of other studies that have described the times for the last three 200 metres sections in Thoroughbred races of 1200–1250 metres distance. In this study the aim was to describe whip use and the horses' performance during races, and investigate associations between racing performance (as judged by ARB stewards) and whip use (again, as judged by ARB stewards). Under the ARB rules, only horses that are in contention can be whipped, so we expect that whippings will be associated with superior performance, and those superior performances will be explained by an effect of whipping on horse velocities in the final 400 m of the race. We were also interested to determine whether performance in the earlier sections of a race predicted whip use in the latter sections of a race.

## Methods

A retrospective inspection of recordings of races was conducted by two experienced stewards at Racing NSW. Both were experienced in counting whips strikes by review of race recordings.

Stewards of Racing NSW administer the Australian Racing Board Rules of racing, and deal with breaches of the Rules, including the whip rules. The Rules describe limits on the number of whip strikes, and the frequency of whip strikes in different sections of the races, and they are therefore required to count the whip strikes in different sections of races.

In racing, penetrometers are used to assess the resistance of the turf as a racing substrate and the depth to which they penetrate the surface of the track is usually expressed in centimetres. A set of penetrometer readings is taken on the morning of race day to gauge the amount of give in the track, but each race-course in Australia has its own system of penetrometer ratings and so cannot easily be compared to one another. Instead, each set of course-specific penetrometer readings for the day is used to assist stewards when rating the track. Tracks are rated from 1–10, with 1 being fastest and 10 being the wettest (see Table 1 [8]).

**Table 1:** Track rating scale used in Australian Thoroughbred racing.

Category	Scale	Description
FAST	1	A dry hard track
GOOD	2	A firm track
GOOD	3	Ideal track with some give
DEAD	4	Track with give, better side of Dead
DEAD	5	Significant amount of give, worse side of Dead
SLOW	6	A mildly rain affected track, better side of Slow
SLOW	7	Rain affected, worse side of Slow
HEAVY	8	Soft track, just into heavy range
HEAVY	9	Very soft, genuine heavy
HEAVY	10	Very soft and wet, heaviest category

Races of 1200–1250 meters (m) distance at a single race track (Canterbury, NSW) were used. The races, all on dry tracks, were chosen by Racing NSW staff. Given that all tracks were dry, stewards' assessments of track condition would have been limited to the categories 1–5 listed in Table 1.

Races with a pre-determined range of similar classes of horse were selected. Penetrometer and stewards' ratings of the track conditions were described. This study was conducted under the approval of the University of Sydney Human Research Ethics Committee (approval number: 11-2009/12299). The authors did not participate in the selection process, other than confirming inclusion and exclusion criteria beforehand.

The attributes of the field and racetrack conditions in the 5 races selected for study appear in Table 2.

**Table 2:** The attributes of the field and racetrack conditions in the 5 races selected for study.

	Race 1	Race 2	Race 3	Race 4	Race 5
Distance (m)	1250	1250	1250	1250	1200
Race Starters	11	14	13	11	12
Eligible horses	9	11	10	7	11
Penetrometer	4.47	4.47	Mv*	4.62	4.62
Steward's Track rating	2	2	2	3	3
Winning time (s)	73.61	72.68	73.68	72.95	69.28

There were 61 starters in five races, and there were 13 horses ineligible because they did not meet one or more of the criteria for inclusion in the analyses.

*Mv = Missing value.

For the purposes of this study, we were interested only in whip use that aligned with the rules of racing. Horses were excluded if:

1. Data for that horse in another race had already been included.
2. The horse was subject to a post-race veterinary examination.
3. The stewards noted poor horse performance in the race.
4. Their performance was subject to any stewards' enquiry.
5. If the stewards noted that the horse's race performance was unusual, for example, going unusually fast early in the race, or being blocked for a run.
6. The stewards noted that interference by another runner had compromised the horse's performance in the race.
7. The jockey was charged with an offence under Australian Racing Board rules of racing* related to use of whips.
8. The horse was whipped before the 400 m mark.

*The whip rules applicable at the time were AR137A (1–5, 7–9, as set out pages 62–63 of the Australian Racing Board Rules, 26 September 2009 [4]).

### Measurements

Times for three 200 m sections of each race were recorded with an electronic sensor on each horse. These sections were from the 600, 400 and 200 m positions from the finish (S3, S2 and S1)) Forehand and backhand whip strikes to the hindquarters with a padded whip (specifications available from Racing Victoria [9]) were identified and counted for the race section 400 to 200 m from the finish (S2), and the section 200 m to the finish (S1). As is the case in the ARB stewards' current practice, slaps, a whip and/or hand motion on the neck or shoulders with the jockey's hands on the reins, were not included in the counts. “Slaps” are not subject to regulation in the Australian Racing Board rules of racing.

Times for the 200 m sections from the 600, 400 and 200 m positions were derived from an electronic timing system that used underground transmitters and a receiver in each horse's saddle cloth. The timing system also reported each horse's placing at the 600, 400, 200 m, and at the finish.

### Statistics

Performances were described as total race times, 400 m, 200 m and final race placing and sectional times for S3, S2 and S1. A split-plot repeated measures analysis of variance with a residual maximum likelihood (REML) algorithm was used to compare the three sectional times in the five races. The REML method allows for the possibility of a change in the variance across sections. Relationships between whip strikes and racing performance over the last 400 m of the race were examined using stepwise regression analyses. Stepwise logistic regression analysis was used to investigate potential predictors of the probability of a horse finishing in the first 3 places (Place-123). Whip counts and sectional times were used in this analysis.

We used a stepwise regression to investigate potential predictors of a horse's final placing. Predictors used were the number of starters in a race, race distance, horse placing at the 400 m and 200 m marks, the sectional and total number of whips between 400 m-200 m and 200 m-finish.

We used a test criterion value of 1.0 in the stepwise regression analyses. This value determines whether a predictor is included or excluded in a model, and corresponds to a P value of about 0.3. However, the predictors that were included in the final analysis were generally significant with a P value of less than 0.05. Strengths of association were described using adjusted R^2^.

## Results

There were 61 starters in five races, and there were 13 horses ineligible because they did not meet one or more of the criteria for inclusion in the analyses. 47/48 (98%) of horses were whipped in this study, indicating that the overwhelming majority were “in contention”. It is worth noting that no horse in this study was guilty of a breach of R.137(b). This implies that, in the opinion of the stewards, all horses included in this study were ridden in a manner that maximised their opportunity of good performance.

Table 3 presents the sectional times and whip counts in the three final 200 m sections. Only 24 horses (50%) were whipped in Section 2.

**Table 3:** Sectional time means (± standard deviations; SD), and median numbers (and ranges) of forehand and backhand whip strikes in the 600 to 400 m (S3), 400 to 200 m (S2) and 200 m to finish (S1) sections.

Section	S3	S2	S1
Section time (s)(± S.D.)	11.64*(0.27)	11.77∧(±0.28)	12.14∧(±0.43)
Forehand whips(range)	0(0)	0(0)	0(0–11)
Backhand whips(range)	0(0)	0.5(0–5)	5.5(0–14)
Total whips(range)	0(0)	0.5(0–5)	6(0–14)

*n = 48;

∧ n = 47.

**Figure 1:**
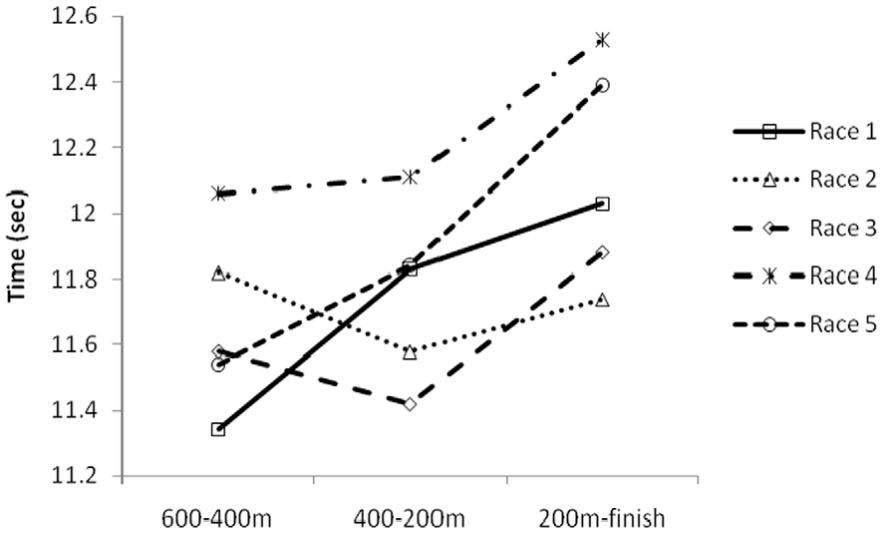
Times for three 200 m sections in 5 races.

From the REML analysis of section times the model with a variance changing across sections was significantly better than a constant variance model (P<0.001). The estimates of variance were 0.014 for S3, 0.028 for S2 and 0.113 for S1, representing an 8-fold increase in variance from S3 to S1. Overall, the mean time for S1 was significantly greater than for S3 (P<0.001) and S2 (P<0.001), indicating reduced velocity in the final section. Section 3 mean time was also significantly less than S2 mean time (P<0.001). There was also a significant interaction between race number and sectional times, (P<0.001). These effects remained after removal of the 1200 m race from the analyses. Figure 1 illustrates the times for the three 200 m sections from the 600, 400 and 200 m positions.

There was a weak but significant association between placing of the horse at the 400 m position, and the combination of total starters in the race and total whip strikes from the 400 m to the finish (P = 0.01, adjusted R^2^ = 10.2%). There were, on average, 0.40 more whips between 400 m and the finish line for a horse placed one ahead at the 400 m position. This relationship was described by the following equation:
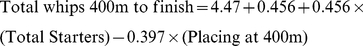



There was also a significant association between placing of the horse at the 200 m position, and subsequent whip use between the 200 m position and the finish. There were, on average, 0.44 more whips between 200 m and the finish line for a horse placed one ahead (P = 0.001, adjusted R^2^ = 17.7%).
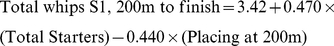



### Predictors of Place-123

The stepwise logistic regression indicated that the best single explanatory variate of the probability of a horse being placed first, second or third was the horse's placing at the 200 m (P = 0.017). Its placing at the 400 m position was also a significant predictor (P = 0.030).
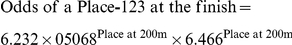



When placings at 400 m and at 200 m are removed from the stepwise regression of the probability of a finish place, the number of whips used in S1 between 200 m and the finish was a significant predictor (P = 0.021), while the number of whips between 400 m and 200 m was not significant (P = 0.810). Neither of the two section times S2 and S1 significantly explained the probability of Place-123 using P<0.05. However, the P value for Section 2 was 0.070.

Table 4 summarises the significant findings.

**Table 4:** Significant correlations between whip counts and performance measurements, and significant predictors of finishing in 1^st^, 2^nd^ or 3^rd^ place (Place-123).

	Whips S1+S2	Whips S1	Place-123
Place at 400	P = 0.01R^2^ = 10.2%		P = 0.03
Place at 200		P = 0.001R^2^ = 17.7%	P = 0.017
Section 2 time			P = 0.07
Section 1 time			ns

(R^2^ = adjusted coefficient of determination, P probability. S1 =  race section from 200 m to finish; S2 =  race section from 400 m to 200 m).

## Discussion

We have presumed that any horses that were whipped when out of contention would have been subject to a stewards' investigation and therefore ineligible for inclusion in the current study. Therefore, we are unsurprised to find that whippings were associated with superior performance and that there was an association between final placing and the number of whip strikes in the final 200 m section. However, there was a significant decrease in velocity in the final 200 m section of the race, when free whipping is condoned for 100 m according to the Australian rules of racing (ARB 2010), and when 98% of horses in the current study were whipped. Fundamentally, the relationship between whipping and placing seems unlikely to be causative. This association more likely reflects the more frequent use of the whip by jockeys after being in a relatively advanced position at 400 m and 200 m from the finish.

This study highlights the challenges stewards face when policing the rules of racing surrounding whip use. In the current study, the one horse that was not whipped at all over the last 400 m was in 9th place out of 13 starters and 10 eligible horses at the 400 m, 4th/10 at the 200 m, and yet finished the race in first place. It is interesting to note that despite being in 9^th^ place out at the 400 m position, this horse was not considered “out of contention”. This speaks of outstanding horsemanship on the part of the winning jockey and appropriate preparation of the horse by the trainer but begs the question: “How is out of contention defined?” Presumably, it should not take into account the performance of the horse over the final 400 m, because the rule expects the jockey to make a decision as to whip or not whip at or after the 400 m position. So, perhaps to be categorised as “out of contention”, horses must be very poorly placed or incapable of galloping normally due to injury or problematic (e.g., erratic) behavior.

There are further challenges for the stewards when policing the requirement that horses “show a response” [to the whip] if whip use is to be considered correct. Our results indicate that, on average, there was no response in terms of velocity over the final 400 m or 200 m distances that significantly influenced the likelihood of finishing in the first three placings. Given the considerable decrease in average velocity during the final 200 m of the race, when horses were fatigued and slowing, it is difficult for stewards to assess responses, either appropriate or inappropriate. Only 5 horses increased velocity in the last two 200 m sections. One of those horses was not whipped in the last 400 m.

Although we have no data on the jockeys' beliefs or their trainers' instructions, the race tactics in races we studied might reflect jockeys' belief that success in a 1200–1250 m race is more likely by achieving a superior relative position at the 400 and 200 m positions, followed by more frequent whip use. That would be a reasonable view by jockeys, given the results in this study. However, riding a horse that is more capable than its competitors of achieving a more prominent, forward position at the 400 and 200 m positions appears to be the critical contributor to superior performances in races of this distance. Horses in superior race positions at the 400 and 200 m were whipped more frequently, and the whipping in the final two 200 m sections had no significant effect on velocity that explained likelihood of racing success. That said, it remains possible that whip use in the final stages of a race really does improve relative performance at a stage when all horses are slowing, but more frequent and sensitive methods of measuring velocity may be required to detect such a cause and effect linkage.

Limitations to this study should be noted. Races were selected on the basis that they fulfilled a number of predetermined criteria and so were not chosen at random. To reduce variability, the study was restricted to one racetrack, and to narrow ranges of race distance, track conditions and penetrometer readings. Results of similar investigation in other racing circumstances, and in races with different prize money, might differ. However, before conducting further studies in other race conditions, it might be more important to further investigate the locomotory responses of racing Thoroughbreds to whipping by jockeys. Is there a locomotory mechanism that could explain a potential causative link between whip use and consequent performance? Such an investigation could consider use of suitable accelerometers and global positioning system (GPS) loggers to study gait and velocities, synchronous with whipping.

Certainly, the use of more sensitive timing technologies merits consideration. The results in the current article were expressed as times for 200 m sections, and as race times, simply because the industry uses these time-based units to describe performances. Velocity during the race should ideally be expressed in metres per second (m/s). However, the use of non-SI units, in this instance, may enhance the relevance of the current findings for industry participants.

Previous studies have demonstrated that fatigue ensues in equine muscle after 800 m of gallop at maximal velocities because adenosinetriphosphate (ATP) and inorganic phosphate accumulate in muscle [10] after that level of exercise. Fatigue is the likely explanation for the decreased running speed in the final two 200 m sections of the race, as is reported here. Success in races we describe here was not dependent on achieving peak speeds at the end of the race. This finding probably reflects jockey riding tactics, an influence that, again, may differ in races of different distances, prize money or in races conducted in different regulatory environments.

Current ARB rules of racing condone free use of the whip in the final 100 m of races. In this study, horses were slowing significantly at that time. Horses were being whipped more when they were slowing. These results also suggest that Thoroughbred racehorses are capable of producing their highest speeds in the last 600 m of a race without whipping. In the races studied, those highest velocities were produced, on average, in the 600 to 400 m sections of the races. Horses that produced superior relative position at the 400 m mark were then hit more frequently, with a correlation with final race outcomes.

Deuel and Lawrence [7] used high-speed cinematography to investigate gait characteristics during “urging” in Quarter horses during gallops at 12.6 m/s. The experimental treatment consisted of the rider “goading the horse with a riding crop” on the leading shoulder approximately once per stride. Urging by the rider had no detectable effect on the average velocity. However, rider urging did cause a significant increase in stride frequency and a decrease in stride length. Results in Thoroughbreds when whipped on the hindquarters during galloping at higher speeds while fatigued may differ. Further investigations of the relationships between whip use and locomotion during Thoroughbred races would be of interest from a biomechanics perspective.

The results in this study do not support a conclusion that whipping cannot affect velocity of an individual Thoroughbred racehorse during the final 400 m section of a race. The absence of a significant prediction of racing success by velocity in the final 200 m section may mask different responses among horses. Highly sensitive, accurate and frequent measurements of velocity and/or position during such whipping in a large number of horses during races could address this issue.

Any effect of whipping on velocity in this study may have been a transitory. Such an effect, if it exists, may differ among horses. However, on average, any such transitory change in velocity did not significantly affect velocity over 400 m enough to change the likelihood of being placed in the first three. A sustained increase in velocity or reduced rate of deceleration due to whipping would have to be explained by an increase in muscle ATP concentration, or effects on fatigue resistance or economy of locomotion. It is difficult to construct a possible mechanism that causally links whipping to a sustained increase in ATP supply by increased oxygen transport or anaerobic glycolysis. On the contrary, the report by Deuel and Lawrence, in Quarter horses, suggests that whipping is likely to change locomotion but not change velocity [7].

To summarise, the results of this study show that jockeys in more advanced placings at the 400 and 200 m positions before the post in races whip their horses more frequently. To gain the advantageous placings at 400 m positions, no horses were whipped while between the 400 and 200 m positions only half were whipped. On average, they achieved highest speeds when there was no whip use, and the increased whip use was most frequent in fatigued horses. That increased whip use was not associated with significant maintenance of velocity as a predictor of superior race placing at the finish of the race. Further studies with on-board sensors of gait characteristics are required to study responses to whipping in individual horses.

The authors conclude that, under an ethical framework that considers costs paid by horses against benefits accrued by humans [11], these data make whipping tired horses in the name of sport very difficult to justify. However, it is worth noting that other ethical frameworks would not condone the practice even if it did, contrary to the findings of this study, cause horses to run faster.
